# Scatter Correction for Heart SPECT images Using TEW method

**DOI:** 10.1120/jacmp.v9i3.2767

**Published:** 2008-06-23

**Authors:** Vahid Changizi, Abbas Takavar, Azadeh Babakhani, Mahdi Sohrabi

**Affiliations:** ^1^ Departments of Radiology Shariati Hospital Tehran Iran; ^2^ Medical Physics Tehran University of Medical Sciences Shariati Hospital Tehran Iran; ^3^ Nuclear Medicine Section Shariati Hospital Tehran Iran

**Keywords:** SPECT, Scatter correction, ^99m^Tc, Triple Energy Window

## Abstract

Radionuclide imaging has the potential to be used in quantitative analysis of the regional function of organs. However, quantification of SPECT images is degraded by many factors such as Compton photon scattering. This could have a destructive effect on clinical reports so it is important to do scatter correction to get better quality SPECT images. We intended to determine how scatter correction with the TEW method can help physicians who look at heart SPECT images, get better reports. This study used the TEW method for scatter correction, which was proposed by Ogawa et al.,^(9)^ using the two narrow windows on either side of the photopeak (20% down and 20% up of the photopeak respectively). Injection of radiopharmaceutical ^99m^Tc was used for medical imaging. In the Shariati Hospital, Tehran, we studied a total of 80 patients with heart disease indications (43 men and 37 women) over the ages of 30–80 years. Contrast and sharpness were considerably improved after scatter correction so physicians could look at defects better. In a few cases scatter correction changed heart defect reports to normal. Using TEW, sensitivity and specificity increased from 86% to 94% and from 61% to 84% respectively. This method was simple to use in clinics.

PACS numbers: 87.57.uh

## I. INTRODUCTION

Single Photon Emission Computed Tomography (SPECT) shows function by means of a three dimensional activity distribution of a radioactive tracer, which was injected prior to the measurement.^1^, ^2^ SPECT images could help if the organ activity is normal. However quantification with SPECT images is degraded by many factors such as Compton photon scattering. When a beam of X‐rays interacts with atoms in a material, two scattering processes occur. Tightly bound electrons are set into oscillation and radiate X‐rays of the same wavelength as that of the incident beam. More loosely bound electrons scatter part of the incident beam and slightly increase its wavelength in the process. The exact amount of increase depends on the scattering angle. The former is called coherent scattering and the latter incoherent or Compton scattering; both types occur simultaneously in all directions. A number of methods have been developed to decrease the Compton problems on SPECT images.

A general idea of the magnitude of scatter in myocardial imaging is an estimate that the ratio of scattered to unscattered (primary) counts, *SP*, is approximately 0.34 for ^99m^Tc and 0.95 for 201Tl.^(3)^ Fujioka et al.,^(4)^ allowed the triple energy window (TEW) method to be applied for Compton scatter correction in conventional SPECT systems without any hardware for TEW acquisition. They used brain phantom and obtained relative activities in white and gray matter with more accuracy. Perisinakis et al.,^(5)^ using double energy window (DEW) and TEW for scatter correction could distinguish lesions in liver studies. Akihiro Kojima et al.,^(6)^ investigated scatter correction in transmission computed tomography (TCT) imaging by the combination of an uncollimated transmission source and a parallel‐hole collimator. They employed the TEW as the scatter correction and found scattered photons transmitting through the object have a peak within the main energy window, based on their phantom studies.

Bai et al.,^(7)^ elucidated the clinical usefulness of scatter correction with an artificial neural network (ANN) in ^99m^Tc and 123I dual‐isotope SPECT. ANN method separated the 123I and ^99m^Tc primary photons well. In a clinical environment Zaidi et al. stated, “researcher must be willing to compare clinics results with and without scatter correction.”^(8)^

On the whole, it is important to do scatter correction to get SPECT images with better quality. Our study focused on scatter correction in the clinic using TEW, as proposed by Ogawa et al.^(9)^ We intended to determine how this method could help physicians who look at heart SPECT images, get better reports.

## II. MATERIALS AND METHODS

This study used the TEW method for scatter correction, as proposed by Ogawa et al.,^(9)^ using the two narrow windows on either side of the photopeak (20% down and 20% up of the photopeak respectively). Equation 1 is applied for each pixel in the photopeak projections to calculate scatter counts.

(1) $$C_{scat}∼(C_{lower}/W_{s}+C_{upper}/W_{s})W_{m}/2$$

Where

$C_{\text{lower}}=\text{counts}\;\text{in}\;\text{left}\;\text{window}$

$C_{\text{upper}}=\text{counts in right window}$

$W_{s}=\text{width of left and right scatter windows}\;(\text{keV})$

$W_{m}=\text{width of photopeak window}\;(\text{keV})$

$C_{\text{scat}}=\text{number of scatter counts}$

The calculated scatter counts were subtracted from total counts for each pixel.

An ADAC Genesys dual head SPECT machine (Philips Medical Systems, Andover, MA) with a low energy high resolution parallel collimator was used. The SPECT acquisition applied a 64×64 matrix and was continuous over 180° in 5° steps for 1 rotation at 800 sec., 36 projections at 25 sec. were being done. A Butterworth processing filter was applied, using a cut off frequency of 45% that was suitable for detecting defects. Image reconstruction was done by a filtered backprojection method and a ramp filter was applied for correction. Injection of radiopharmaceutical ^99m^Tc was used for medical imaging.

In this study a total of 80 patients with heart disease indications (43 men and 37 women) over the ages of 30–80 years old, from Shariati hospital in Tehran, Iran, were studied. All of the images were reviewed before and after scatter correction by three veteran clinical specialists in nuclear medicine. Finally, the angiography results of patients prepared by radiology section were collected as a gold standard. On the basis of angiography results, sensitivity and specificity could be found. Sensitivity is how well the test identifies patients with heart defect. It is the true positive fraction.

Sensitivity gives the proportion of heart defects identified by the TEW for scatter correction relative to all cases that actually have the heart defect. Specificity is the true negative fraction or the ability of the test to identify patients who don't have the heart defect. In this way the test was done directly on patient images for better evaluation of its ability in practice.

## III. RESULTS

**Figure 1:**
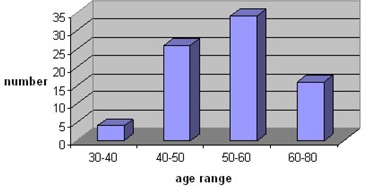
Age distribution of people studied with heart disease indications

**Figure 2:**
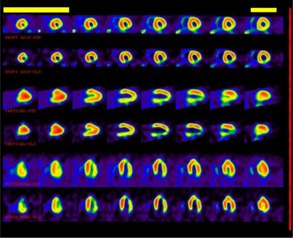
SPECT cardiac images in three positions; short axis, vertical and horizontal ‐ both without scatter correction (first rows) and with scatter correction (second rows).

Fig. 1 shows most of the patients were between 50–60 and 40–50 years old respectively. However, no relationship between the patient age distribution and the results of scatter correction were found. Fig. 2 shows SPECT cardiac images in three positions of short axis, vertical and horizontal both without and with scatter correction. For each position rows 1 and 2 present without and with scatter correction respectively. After scatter correction physicians could identify defects better. Table 1 compares specificity and sensitivity before and after scatter correction. For sensitivity and specificity the gain of 8% and 23% were found respectively.

**Table 1:** Specificity and sensitivity before and after scatter correction

*Type of technique*	*Specificity*	*Sensitivity*
Without TEW	61%	86%
With TEW	84%	94%

## IV. DISCUSSION

In this study it was assumed the spatial distribution of the scatter within two windows on either side is the same as the photopeak window. Kojima et al.^(6)^ also showed that scattered photons transmitted through the object have a peak within the main energy window. Therefore, this study subtracted the Compton scatter windows (side windows) from the main window for scatter correction. From a clinical standpoint it is important to get accurate radionuclide distribution. Fig. 2 showed that the TEW could make it possible. Our method corresponds to Zaidi et al.^(8)^ and stresses the importance of comparing clinic results with and without scatter correction. Much research has been done on this subject using phantoms, but there are few studies in clinics. We found that it is simple to use the TEW in clinics and at the same time increase sensitivity and specificity. By this correction it was found a few scatter sources such as collimator have destructive effect on SPECT images. So after correction, heart defects could be found. Since King MA et al.^(3)^ and Zaidi et al.^(8)^ brought a ratio of 0.34 for scattered to unscattered (primary) counts, increase of contrast and sharpness after correction could be understood.

In similar studies, Perisinakis et al.^(5)^ used the TEW to distinguish lesions in liver. In a few cases heart defect reports were changed to normal after correction. As a matter of fact, the TEW could increase specificity. Table 1 confirms the increase of 23%.

Also, our studies confirmed the results of Fujioka et al.,^(4)^ even though they used brain phantom.

## V. CONCLUSION

Using the TEW method for scatter correction improves physicians' detection of defects in SPECT images. Sensitivity and specificity were increased from 86% to 94% and from 61% to 84% respectively. The TEW method provides a simple method for scatter correction that can be applied in the clinic.

## ACKNOWLEDGMENT

This Study has been supported by Medical Sciences/University of Tehran. My big thanks go to the Nuclear Medicine section of Shariati Hospital.
